# The Influence of Sex and Season on Conspecific Spatial Overlap in a Large, Actively-Foraging Colubrid Snake

**DOI:** 10.1371/journal.pone.0160033

**Published:** 2016-08-04

**Authors:** Javan M. Bauder, David R. Breininger, M. Rebecca Bolt, Michael L. Legare, Christopher L. Jenkins, Betsie B. Rothermel, Kevin McGarigal

**Affiliations:** 1 Department of Environmental Conservation, University of Massachusetts, Amherst, Massachusetts, United States of America; 2 NASA Ecological Programs, Integrated Mission Support Services, Kennedy Space Center, Florida, United States of America; 3 Merritt Island National Wildlife Refuge, Titusville, Florida, United States of America; 4 The Orianne Society, Athens, Georgia, United States of America; 5 Archbold Biological Station, Venus, Florida, United States of America; University of Regina, CANADA

## Abstract

Understanding the factors influencing the degree of spatial overlap among conspecifics is important for understanding multiple ecological processes. Compared to terrestrial carnivores, relatively little is known about the factors influencing conspecific spatial overlap in snakes, although across snake taxa there appears to be substantial variation in conspecific spatial overlap. In this study, we described conspecific spatial overlap of eastern indigo snakes (*Drymarchon couperi*) in peninsular Florida and examined how conspecific spatial overlap varied by sex and season (breeding season vs. non-breeding season). We calculated multiple indices of spatial overlap using 6- and 3-month utilization distributions (UD) of dyads of simultaneously adjacent telemetered snakes. We also measured conspecific UD density values at each telemetry fix and modeled the distribution of those values as a function of overlap type, sex, and season using generalized Pareto distributions. Home range overlap between males and females was significantly greater than overlap between individuals of the same sex and male home ranges often completely contained female home ranges. Male home ranges overlapped little during both seasons, whereas females had higher levels of overlap during the non-breeding season. The spatial patterns observed in our study are consistent with those seen in many mammalian carnivores, in which low male-male overlap and high inter-sexual overlap provides males with greater access to females. We encourage additional research on the influence of prey availability on conspecific spatial overlap in snakes as well as the behavioral mechanisms responsible for maintaining the low levels of overlap we observed.

## Introduction

The nature of interactions among conspecifics has a strong influence on their degree of spatial overlap, which in turn influences multiple ecological processes, including social behaviors [1], mating systems [2], and population density and regulation [3,4]. The degree of spatial overlap among conspecifics can vary widely within and among species [1,5,6,7,8], ranging from extensive overlap to exclusive space use [9]. Patterns of conspecific spatial overlap and the factors influencing those patterns are described for many terrestrial taxa, including mammalian carnivores [1,5,10,11,12] and herbivores [2], small mammals [6,13,14], birds [15], and lizards [16]. However, relatively little is known about the factors influencing spatial overlap in snakes.

Studies on snake movement patterns and space use have reported widely varying levels of spatial overlap, ranging from extensive home range overlap [17,18,19] to low levels of overlap [20,21,22]. Other studies have reported extensive home range overlap but conspecific avoidance at the scale of specific shelters [23,24]. However, active defense of and conspecific exclusion from an area (i.e., territoriality) [9] appears very rare in snakes [25,26,27,28]. Indeed, many species of snakes show very dense conspecific aggregations [25] yet these aggregations often occur near high concentrations of resources such as communal hibernacula, gestation sites, distinct habitats (e.g., wetlands or riparian habitats), cover objects, prey, or potential mates [25,29,30,31]. In such cases, the benefits and efficacy of maintaining exclusive access to those resources may be far below the costs [32] although the only two studies demonstrating territorial behavior in snakes both involved spatially clustered resources, i.e., sea turtle nests [27] and shelter sites [28]. Excluding individuals from an area where resources are widely dispersed may prove similarly uneconomical. Nevertheless, despite the variability in patterns of spatial overlap reported for snakes, most studies reporting information on inter-individual home range overlap in snakes merely report population-level summary statistics and do not examine how overlap varied temporally or by sex. Describing patterns of home range overlap within and between sexes and how those patterns vary seasonally may provide insights into the mechanisms driving the degree of observed overlap.

The eastern indigo snake (*Drymarchon couperi*), an endemic of the southeastern coastal plain of the U.S.A., is the longest native snake in North America, regularly exceeding 2 m in snout-vent length [33,34,35]. This species is an active forager that feeds year-round on a diversity of prey [36,37]. Additionally, *D*. *couperi* home ranges and daily movement distances are among the largest reported for terrestrial snakes, with males maintaining larger home ranges on average than females [38,39,40]. Most *D*. *couperi* breeding activity occurs from October–March, during which males search for females and may engage in male-male combat [34,38,40,41]. In the northern portion of its range (i.e., southern Georgia), *D*. *couperi* have relatively small winter home ranges (< ≈ 10 ha) corresponding to their near-exclusive use of xeric sandhills supporting gopher tortoise (*Gopherus polyphemus*) burrows for winter refugia [34,40,42]. During the spring–fall, home range size can increase over 25-fold, with males having larger home ranges [40]. In contrast, in the central portion of its range (i.e., central peninsular Florida), *D*. *couperi* maintains smaller yet seasonally-invariant year-round home ranges (mean of 149.12 ha for males and 48.97 ha for females) with the exception that males generally increased their home range sizes during the breeding season, presumably because of mate-searching movements [38].

Despite our increasing understanding of *D*. *couperi* spatial ecology, very little is known about how individual home ranges overlap spatially or what factors may affect the degree of overlap. Hyslop et al. [40] reported that several *D*. *couperi* in southern Georgia had overlapping year-round home ranges. No studies have to-date discussed inter-individual home range overlap for *D*. *couperi* in peninsular Florida. While Bauder et al. [38] described intra-individual home range overlap they did not examine inter-individual home range overlap. In this paper, we describe inter-individual spatial overlap of *D*. *couperi* in peninsular Florida and examine how the degree of overlap varied by sex and season (breeding season vs. non-breeding season).

## Materials and Methods

### Ethics Statement

All researchers adhered to the Guidelines for Use of Live Amphibians and Reptiles in Field and Laboratory Research published by the American Society of Ichthyologists and Herpetologists. All surgeries to implant radio transmitters were conducted by professional veterinarians experienced in this procedure and isoflurane was used as an anesthesia in accordance with approved protocols. All work was approved under permits from the appropriate agencies and institutions, including United States Fish and Wildlife Service (TE28025A-1), Florida Fish and Wildlife Conservation Commission (WX97328), University of Florida Institutional Animal Care and Use Committee (200903450), and Archbold Biological Station Institutional Animal Care and Use Committee (ABS-AUP-002-R).

### Study site and data collection

We monitored *D*. *couperi* using radio telemetry as part of two studies across central Florida. Detailed descriptions of study areas and sampling methodology are described elsewhere but we provide brief descriptions here [38,43]. The first study occurred from 2011–2013 in Highlands County, Florida (27°17ʹ N, 81°21ʹ W). This study area covered approximately 40 km of the Lake Wales Ridge and included a mosaic of public and privately owned lands with a diversity of natural and anthropogenic habitats including scrub, scrubby flatwoods, mesic flatwoods, forested and non-forested wetlands, cattle ranches, citrus groves, and rural and urban development. Additional descriptions of this study area are provided elsewhere [44,45]. Bauder and Barnhart [43] described the procedures for capture, surgical implantation of transmitters, and radio telemetry. We visually confirmed the location of our telemetered snakes for 96.5% of our telemetry fixes and marked those locations with a handheld GPS unit. For the remaining fixes (113 of 3,219), we used triangulation to estimate the snake’s location. We predicted the linear error of these locations following Bauder and Barnhart [43] and retained locations with predicted linear error ≤ 150 m because this degree of error relative to our home range sizes was predicted to still produce accurate and unbiased home range estimates [46]. The second study occurred from 1998–2003 at three study sites throughout central Florida as described in Breininger et al. [39]: Brevard (28°38ʹ N, 80°42ʹ W), Indian River (27°50ʹ N, 80°35ʹ W), and Polk Counties (27°37ʹ N, 81°19ʹ W). These areas also included a diversity of public and private land ownerships. Habitats included scrub, scrubby flatwoods, mesic flatwoods, mesic and xeric hammocks, wetlands, coastal scrub, mangrove, and rural and urban development. Breininger et al. [39] provide descriptions of the procedures for surgical implantation of transmitters, and radio telemetry. We hereafter refer to these two datasets as Highlands and Brevard, respectively. In both studies, ≥ 90% of captures were opportunistically made during other field activities or while tracking other telemetered snakes. A total of 32 and 103 snakes were captured in each study, respectively, although not all individuals were included in our analyses (see below).

### Home range estimation

Following Bauder et al. [47] we estimated 6-month home ranges for *D*. *couperi* breeding (April–September) and non-breeding seasons (October–March), which coincided with our observations of *D*. *couperi* breeding activity in our study and previously published data [34,40]. We estimated 6-month home ranges for individuals tracked ≥ 105 days (approximately 3.5 months) because data collected over these durations produce unbiased home range estimates [47]. We included data from two Highlands snakes that exhibited complications with the transmitter implantation site or extreme weight loss (≥ 31%) because they exhibited spatial overlap with other telemetered snakes and gave no indication that their space use patterns differed from those of other telemetered snakes. We likewise included data from a single Brevard snake that died from receiving an antibiotic combined with ivermectin during surgery.

We estimated home ranges using 95% fixed kernel utilization distributions (UD) using the plug-in and reference bandwidths with unconstrained bandwidth matrices [48]. We also estimated core areas using the 50% UD. Home range size using both estimators was highly correlated (*r*_s_ ≥ 0.97). However, the reference bandwidth imposed a higher degree of smoothing, resulting in larger home range estimates that allowed more adjacent home range dyads to meet our criteria for adjacency (see below). Additionally, in a study such as ours, where the degree of inter-individual interaction may be under-represented due to infrequent sampling, imposing a higher degree of smoothing for home range estimation may be advantageous, because it incorporates areas where space use may have occurred but was not detected. We therefore used the reference bandwidth matrix. We estimated the bandwidth matrix using the R package ks v. 1.9.2 [49,50].

Because some of the Brevard home ranges had as few as 10 fixes, we created area-observation plots for all home ranges and retained those whose plots reached an asymptote [51,52]. We generated 500 bootstrapped home range estimates for each number of fixes from 5 to *n* where *n* = the total number of fixes for that home range. We considered an estimate to have reached an asymptote if the mean bootstrapped home range size for at least the last 50% of subsampled fixes were within 10% of the home range size estimated with all fixes [38]. However, this relatively conservative criterion excluded several home ranges, including some which visually appeared to reach an asymptote. Because inter-individual variability in home range sizes is often the single greatest source of variability in home range data sets, it is often advantageous to maximize the number of individuals included in an analysis [53]. We therefore reran our area-observation plots using a more liberal criterion defining an asymptote as a mean bootstrapped home range size within 10% of the full home range size for the last five subsampled fixes [52]. We ran all subsequent analyses with both data sets and obtained similar results so we report those using the more liberal criterion.

### Home range overlap

To quantify spatial overlap among individuals, we identified dyads of simultaneously adjacent home ranges, defined as home ranges within the same season with overlapping 99% UD volume contours. This ensured that individuals in the same dyad were tracked during the same temporal (season) and spatial (overlapping UD) extent. We used the 99% volume contour to define adjacency because it approximates the maximum possible area over which an individual could have moved and therefore interacted with conspecifics. We measured home range and core area overlap using the volume of intersection (VI) and utilization distribution overlap index (UDOI) [54,55]. We also calculated the distance between home range centroids for each dyad, where the centroid was the mean x and y coordinates across an individual’s telemetry fixes. However, small home ranges mostly or completely overlapped by larger home ranges had low VI and UDOI despite high degrees of overlap. Therefore, for each dyad, we calculated the probability of each individual occurring within the other individual’s home range (PHR) [54] which is analogous to the proportion of home range *i* overlapped by home range *j* but accounts for non-uniform space use within the home range by using the UD. Because PHR is calculated for each individual in the dyad we used the maximum of the two values (PHR_max_) in all analyses. The higher the PHR_max_ value, the more one home range was contained within the other home range.

We analyzed inter-individual UD overlap for home ranges and core areas separately using a permutation-based multivariate analysis of variance of distance matrices [56,57]. This accounted for both the non-normal distribution of our data and the lack of independence among dyads due to the presence of individuals within more than one dyad. We specified our data as a Euclidean distance matrix upon which the sums of squares was then partitioned between within- and among-group variance in a manner analogous to a parametric analysis of variance. We used 10,000 permutations to calculate exact *P* values with the adonis function in the R package vegan v. 2.2–1 [58]. We tested for an interactive effect of sex (male-female, female-female, and male-male) and season (breeding and non-breeding) on UD overlap and combined Highlands and Brevard data because of sample size limitations. If the initial test was significant, we then conducted pairwise tests using the adonis function within the significant factors and reported adjusted *P* values using sequential Bonferroni corrections [59]. Because of sample size limitations we used an uncorrected α = 0.10.

### Individual use of conspecific space

While the above metrics quantify the degree of spatial overlap at the scale of the entire home range, we were primarily interested in describing the degree to which an individual uses space within a conspecific’s simultaneously adjacent home range. Because the UD provides a probabilistic measure of space use, where each underlying pixel has a probability density value proportional to its expected probability of use, the distribution of conspecific UD densities at an individual’s telemetry fix describes the manner in which that individual utilized the conspecific’s home range. However, our observations of simultaneously adjacent home range overlap only included two, occasionally three, individuals. Because we did not simultaneously monitor all adjacent conspecifics, we felt that only fixes within some “zone of interaction” (ZOI), instead of all fixes, had the potential to be influenced by a conspecific. We defined the ZOI using a two-step process. First, we calculated the 99% UD volume contours for the focal individual and its simultaneously adjacent conspecifics and considered all fixes within the area of overlap as within the ZOI. For fixes outside of the area of overlap, we measured the Euclidean distance to the edge of the focal individual’s 99% volume contour (dist_focal_) and the edges of the 99% volume contours of the conspecifics (dist_consp_). All fixes where dist_consp_ ≤ dist_focal_ and that overlapped the conspecific UD were also considered within the ZOI. For all fixes within the ZOI we measured the density values of the conspecific UD (D_consp_) as the density at that fix multiplied by the area of the pixel. We used a constant pixel size (15 × 15 m) for all individuals. We assumed the presence of two conspecifics would represent an additive effect and therefore added the UD when multiple simultaneously adjacent conspecifics were present. We measured D_consp_ twice, once using only simultaneously adjacent individuals of the same sex (i.e., male-male or female-female overlap) and once using only simultaneously adjacent individuals of the opposite sex (i.e., male-female or female-male).

Because the distribution of D_consp_ was highly right skewed, we modeled our data using a generalized Pareto distribution (GPD) in the R package texmex v. 2.1 [60]. We again combined the Highlands and Brevard data because of small sample sizes. The GPD has two parameters, shape and scale, and we modeled both parameters as a function of overlap type, sex, season, and their respective interactions. Overlap type included same-sex (i.e., male-male or female-female) or opposite-sex (i.e., male-female or female-male) overlap. Male-female overlap represents male use of female home ranges while female-male overlap represents female use of male home ranges. Sex was a binary variable representing male use of conspecific space or female use of conspecific space. Season was also binary representing breeding or non-breeding seasons. Because data sparseness prevented us from fitting the global model with all possible interactions, we considered interactive terms of two variables with an additive effect of the third variable. We compared models using the Akaike Information Criterion adjusted for small sample sizes (AIC_c_) [61].

## Results

### Radio telemetry

We estimated 6-month home ranges from 41 Brevard and 16 Highlands snakes that were simultaneously adjacent with at least one other conspecific (Table 1). We obtained 61 conspecific 6-month home range dyads, including 36 male-female, 8 female-female, and 17 male-male dyads. All individuals were considered adults (snout-vent length [SVL] ≥ 122 cm, [34]) and we did not distinguish between gravid and non-gravid females because available data suggest that female *D*. *couperi* reproduce annually [62,63]. Males were significantly longer (SVL [mean ± SE]: males = 168.75 cm ± 3.90, females = 156.69 cm ± 3.53, *t* = -2.29, *P* = 0.0252) and heavier (males = 1.66 kg ± 0.09, females = 1.20 kg ± 0.07, *t* = -4.17, *P* < 0.0001) than females.

**Table 1:** Number of individuals, home range dyads, home range sizes, and tracking intensities for radio telemetered eastern indigo snakes (*Drymarchon couperi*) used in the analyses of conspecific spatial overlap.

	Mean home range size (ha)							
	Males			Females			Mean number of fixes	Mean tracking duration (days)
	Snakes (*n*)	Breeding	Non-breeding	Snakes (*n*)	Breeding	Non-breeding		
		313.68	183.24		36.99	87.3		
6-month		(238.06)	(56.83)		(33.11)	(62.47)		
Highlands	9	*n* = 5	*n* = 8	7	*n* = 7	*n* = 4	57 (10)	163 (17)
		426.68	235.00		84.51	146.26		
6-month		(313.06)	(174.63)		(72.91)	(120.81)		
Brevard	22	*n* = 29	*n* = 27	19	*n* = 19	*n* = 21	19 (4)	157 (22)
6-month		410.07	223.17		71.72	136.82		
Brevard &		(302.78)	(156.44)		(67.40)	(114.61)		
Highlands	31	*n* = 34	*n* = 35	26	*n* = 26	*n* = 25	26 (16)	158 (21)

The mean and standard deviation of home range size, number of fixes, and tracking duration are reported. Sample sizes within each home range column are the number of simultaneously adjacent home range dyads (pooled across sexes). The maximum possible number of tracking days was 183 days for 6-month home ranges.

### Home range overlap

**Fig 1:**
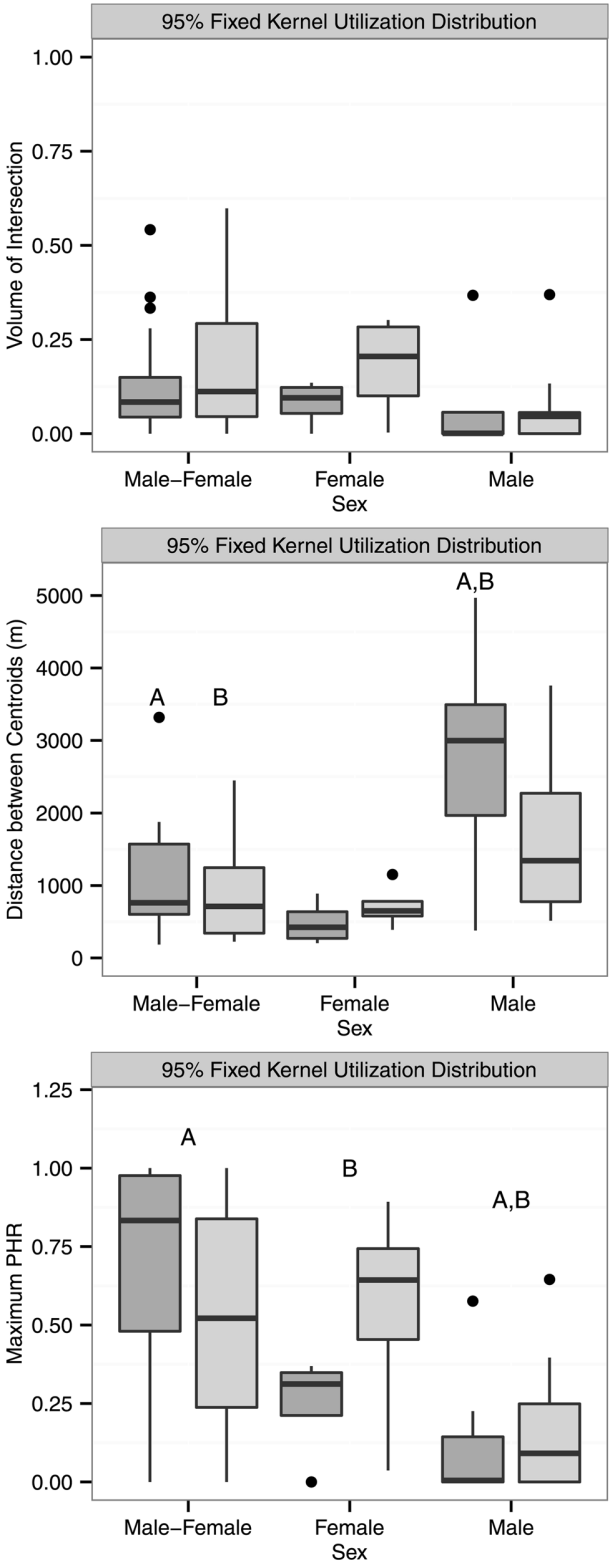
Boxplots of conspecific home range overlap (95% fixed kernel utilization distribution) for simultaneously adjacent eastern indigo snake (*Drymarchon couperi*) 6-month home range dyads (Highlands and Brevard data combined). Home ranges were estimated using an unconstrained reference bandwidth matrix. The thick horizontal line indicates the median, the edges of the boxes the 25^th^ and 75^th^ percentiles, and the whiskers approximate a 95% confidence interval. Female-female dyads are denoted as “Female,” male-male dyads as “Male,” and male-female dyads as “Male-Female.” Breeding seasons (October–March) are denoted with dark gray and non-breeding seasons (April–September) with light gray. Maximum PHR is the maximum probability of home range overlap for each dyad. Pairs of dyads marked with the same upper-case letters (e.g., A, B) are significantly different (*P* <0.10). All pairwise *P* values were adjusted using Holm’s (1979) method. Pairwise comparisons in maximum PHR indicate a significant effect of sex; there was no significant effect of season or sex*season.

Volume of intersection was highly correlated with UDOI for both home ranges (UD) and core areas (50% UD) (*r*_s_ ≥ 0.94, *P* < 0.0001) so we only report the results using VI. Mean home range VI was 0.13 (range = 0.00–0.60) across all dyads. There was no significant effect of sex (*F*_2,60_ = 2.15, *P* = 0.1244), season (F_1,60_ = 1.49, *P* = 0.2365), or their interaction (F_2,60_ = 0.22, *P* = 0.8027) on home range VI. However, there was a significant effect of sex (F_2,60_ = 12.77, *P* = 0.0002), season (F_1,60_ = 2.93, *P* = 0.0904) and sex*season (F_2,60_ = 2.44, *P* = 0.0945) on distance between home range centroids. Following corrections for pairwise error, the distance between breeding season male-male centroids was significantly greater than the distance between male-female centroids during both the breeding (*P* = 0.0126) and non-breeding seasons (*P* = 0.0045, Fig 1). Sex was also significant for home range PHR_max_ (F_2,60_ = 11.55, *P* < 0.0001) but not for season (F_1,60_ = 0.10, *P* = 0.7453) or sex*season (F_2,60_ = 1.88, *P* = 0.1502). Male-male home range dyads had significantly lower PHR_max_ than male-female (*P* = 0.0003) and female-female dyads (*P* = 0.0528, Fig 1).

### Core area overlap

**Fig 2:**
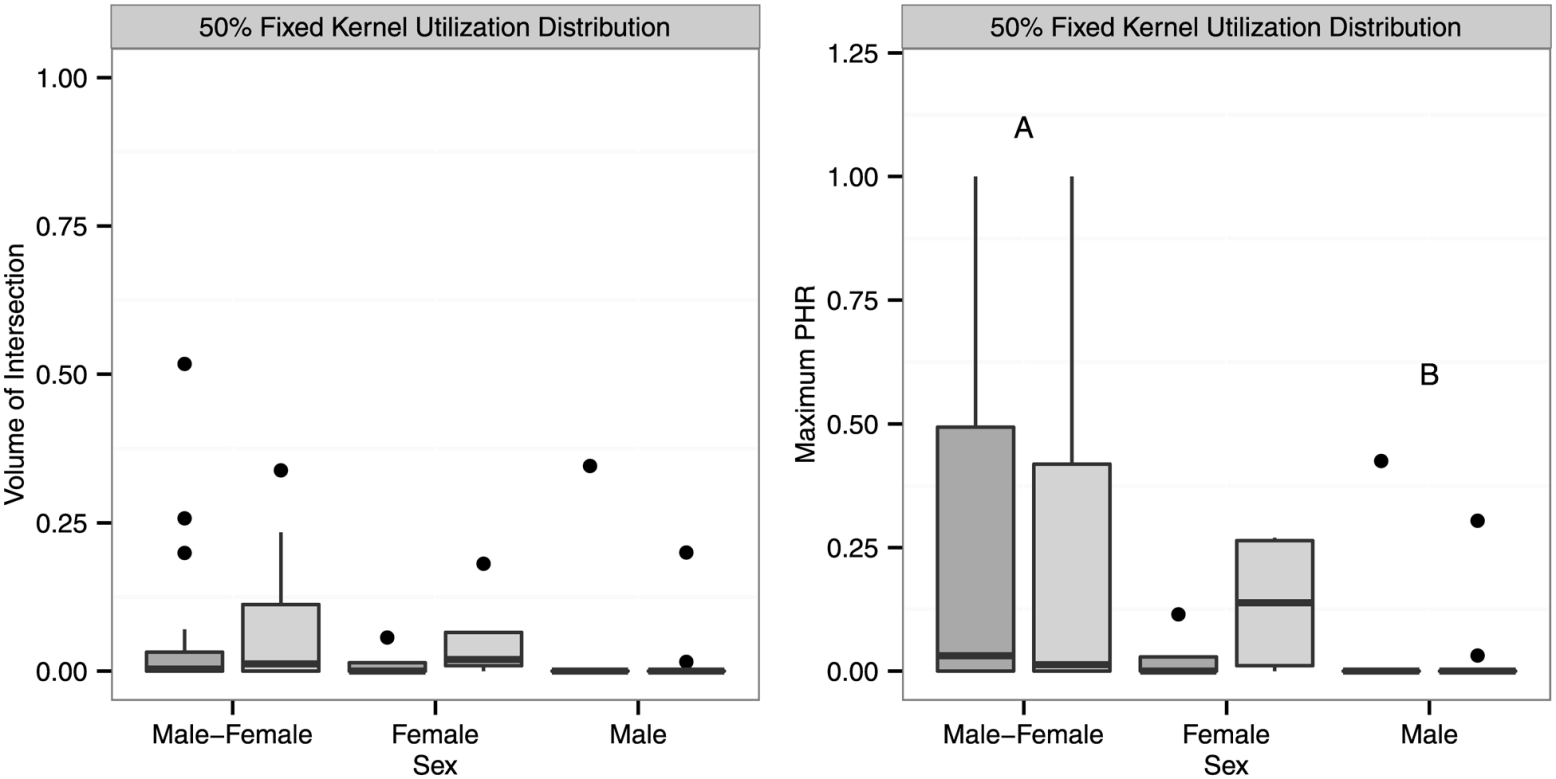
Boxplots of conspecific core area overlap (50% fixed kernel utilization distribution) for simultaneously adjacent eastern indigo snake (*Drymarchon couperi*) 6-month home range dyads (Highlands and Brevard data combined). Home ranges were estimated using an unconstrained reference bandwidth matrix. The thick horizontal line indicates the median, the edges of the boxes the 25^th^ and 75^th^ percentiles, and the whiskers approximate a 95% confidence interval. Female-female dyads are denoted as “Female,” male-male dyads as “Male,” and male-female dyads as “Male-Female.” Breeding seasons (October–March) are denoted with dark gray and non-breeding seasons (April–September) with light gray. Maximum PHR is the maximum probability of home range overlap for each dyad. Pairs of dyads marked with the same upper-case letters (e.g., A, B) are significantly different (*P* < 0.10). All pairwise *P* values were adjusted using Holm’s (1979) method. Pairwise comparisons in maximum PHR indicate a significant effect of sex; there was no significant effect of season or sex*season.

Mean core area VI was 0.05 (range = 0.00–0.52). There was no significant effect of sex (*F*_2,60_ = 0.65, *P* = 0.5474), season (F_1,60_ = 0.06, *P* = 0.8164), or their interaction (F_2,60_ = 0.23, *P* = 0.7909) on core area VI. Sex was significant for core area PHR_max_ (F_2,60_ = 3.27, *P* = 0.0421) but not for season (F_1,60_ = 0.16, *P* = 0.6926) or sex*season (F_2,60_ = 0.32, *P* = 0.7209). The core area PHR_max_ for male-male and male-female dyads were significantly different (*P* = 0.0753, Fig 2).

### Individual use of conspecific space

**Fig 3:**
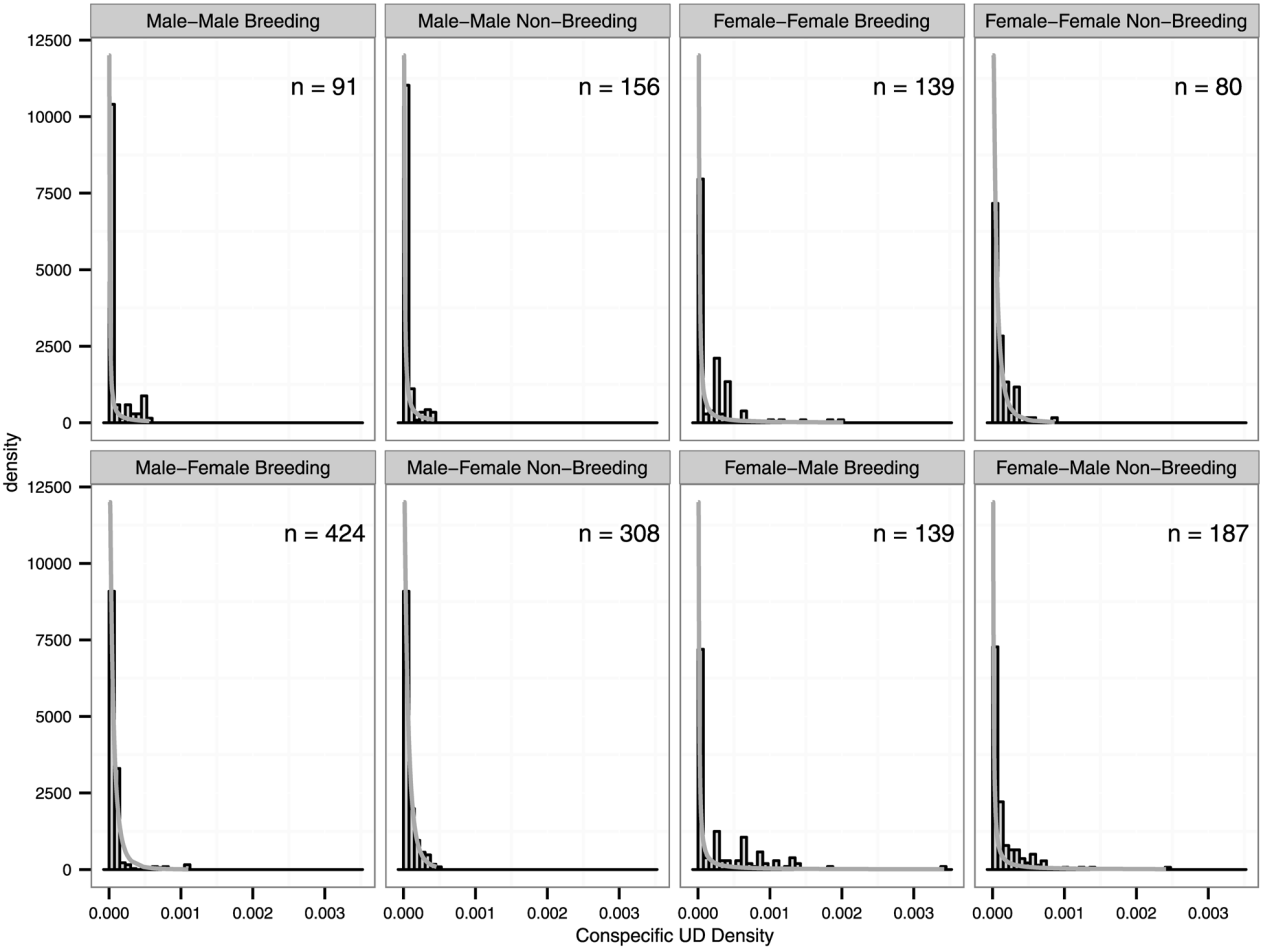
Distributions of conspecific utilization distribution (UD) densities at eastern indigo snake (*Drymarchon couperi*) radio telemetry locations fit using a generalized Pareto distribution. Utilization distributions were calculated using the unconstrained reference bandwidth for each 6-month season (breeding = October–March, non-breeding = April–September). Male-female overlap represents male use of female UD while female-male overlap represents female use of male UD. The number of fixes for each season*overlap type combination are displayed in each panel.

The model including an interactive effect of season and overlap type and an additive effect of sex for shape and scale received all the model support among GPD models for individual use of conspecific space (Table 2). Males used less of other males’ home ranges compared to female use of other females’ home ranges, particularly during the non-breeding season (Fig 3). Female use of male home ranges was greatest during the breeding season, while male use of female home ranges was lower and more temporally consistent.

**Table 2:** Model selection results using a generalized Pareto distribution (GPD) to model conspecific UD density within the zone-of-interaction for 6-month home ranges estimated using the unconstrained reference bandwidth.

Model	Dev	*k*	AIC_c_	ΔAIC_c_	w_i_
φ(Season*Type + Sex), ξ(Season*Type + Sex)	12969.78	10	-25919.41	0.00	1.00
φ(Sex*Type + Season), ξ(Season*Type + Season)	12951.87	10	-25883.60	35.81	0.00
φ(Sex*Type), ξ(Sex*Type)	12948.56	8	-25881.02	38.39	0.00
φ(Sex*Season + Type), ξ(Sex*Season + Type)	12949.54	10	-25878.94	40.47	0.00
φ(Sex + Type), ξ(Sex + Type)	12934.65	6	-25857.25	62.16	0.00
φ(Sex*Season), ξ(Sex*Season)	12899.49	8	-25782.88	136.53	0.00
φ(Sex + Season), ξ(Sex + Season)	12894.01	6	-25775.97	143.43	0.00
φ(Sex), ξ(Sex)	12889.27	4	-25770.52	148.89	0.00
φ(Season*Type), ξ(Season*Type)	12840.95	8	-25665.80	253.60	0.00
φ(Type), ξ(Type)	12835.14	4	-25662.26	257.15	0.00
φ(Season + Type), ξ(Season + Type)	12836.11	6	-25660.17	259.24	0.00
φ(Season), ξ(Season)	12679.46	4	-25350.88	568.52	0.00
φ(.), ξ(.)	12676.76	2	-25349.51	569.89	0.00

The GPD has two parameters, scale (estimated here as log(scale), φ) and shape (ξ), which were both modeled as a function of season (breeding = October–March, non-breeding = April–September), sex, and overlap type (same-sex or opposite-sex). Additive effects (+) were included where model convergence would not permit interactive effects (*). Deviance (Dev) is -2*log-likelihood, *k* is the number of model parameters, and w_i_ = AIC_c_ model weights.

## Discussion

**Fig 4:**
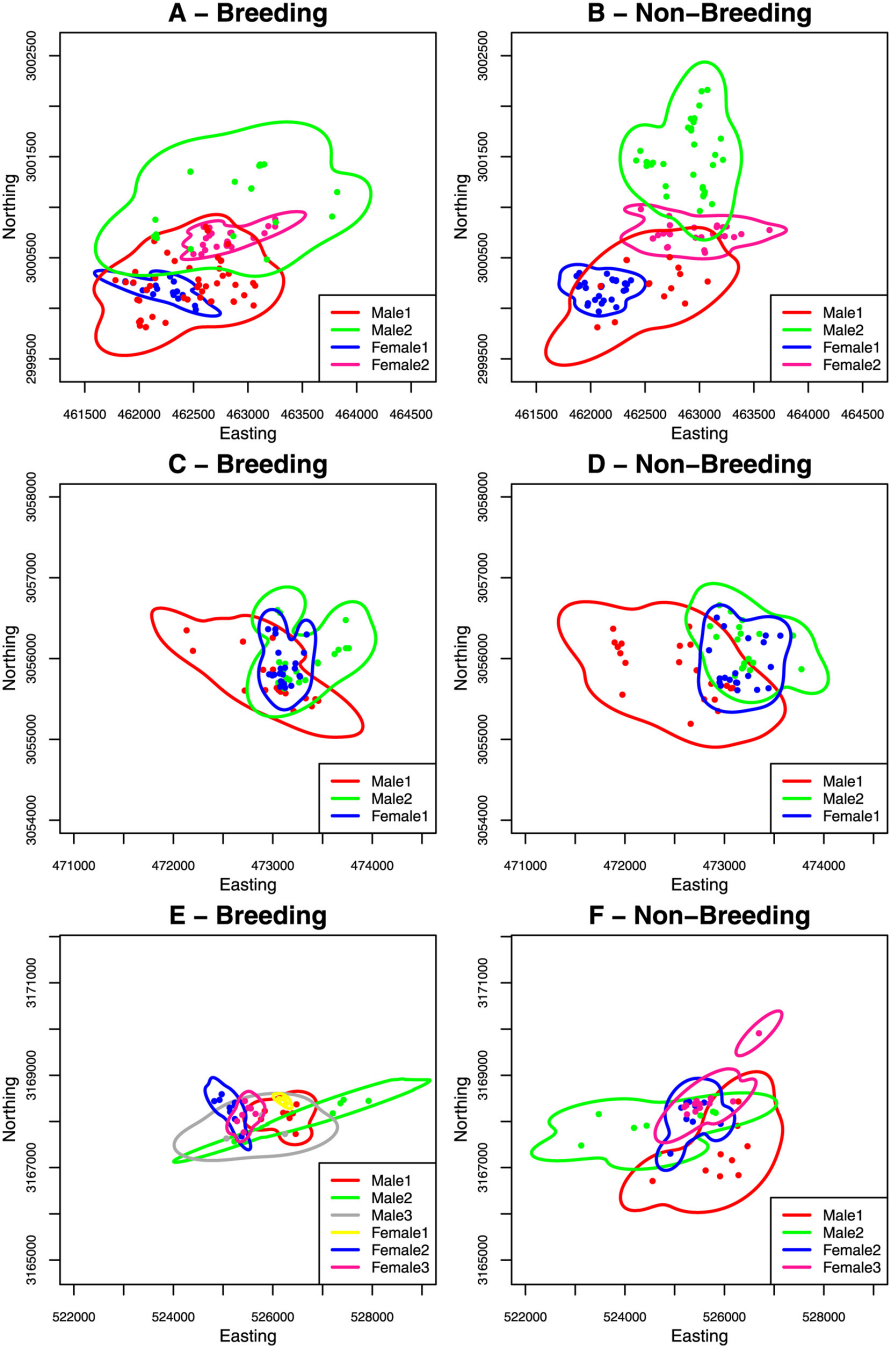
Six-month home range overlap among simultaneously adjacent male and female eastern indigo snakes (*Drymarchon couperi*) for consecutive breeding (October–March) and non-breeding (April–September) seasons from Highlands County, Florida. Panels A and B, C and D, and E and F depict the same individuals. Panels A and B depict an example of reduced male-male overlap between the breeding and non-breeding season. Panels C and D depict the maximum observed male-male overlap during both the breeding and non-breeding season. Panels E and F depict an example of high female-female overlap. Note that not all of the home range estimates depicted here met our criteria for inclusion in the statistical analyses (i.e., were not monitored for ≥ 105 days and/or home range size did not asymptote). Individuals depicted in panel E but not F were lost due to transmitter removal/failure or mortality.

Our study suggests that *D*. *couperi* in peninsular Florida maintain generally low levels of spatial overlap but that the degree of overlap varies interactively by sex and, to a lesser extent, season. Male home ranges would often mostly or completely overlap one or more female home ranges, whereas male-male and, to a lesser extent, female-female overlap was much less (Figs 1, 2 & 4). These patterns persisted when examining core area overlap as only three of 17 (18%) male-male dyads had overlapping core areas (50% UD) compared to four of eight (50%) female-female and 20 of 36 male-female dyads (56%). The probability of occurring within a conspecific’s home range (PHR_max_) was significantly greater for male-female dyads compared to female-female or male-male dyads (Fig 1). This pattern persisted, albeit at reduced levels, when examining core area overlap as PHR_max_ differed significantly between male-male and male-female dyads. There was also evidence for increasing male-female overlap during the breeding season and, while this trend was not statistically significant, it was consistent with our expectations based on *D*. *couperi* mating systems. Webb and Shine [21] report similar patterns with regards to male-male home range overlap in broad-headed snakes (*Hoplocephalus bungaroides*). During the spring and early summer, when individuals inhabited rocky outcrops, male home ranges showed virtually no overlap while female home ranges were contained within male home ranges. Home range overlap was greater within and between sexes during the summer, when individuals moved into adjacent woodlands, but individuals appeared to avoid conspecifics of either sex temporally. Steen and Smith [22] reported low annual home range overlap for eastern kingsnakes (*Lampropeltis g*. *getula*) and also found that male-female overlap was higher than male-male overlap. Similarly, Cottone and Bauer [20] reported that female home ranges were often contained within male home ranges during the breeding season for rhombic skaapstekers (*Psammophylax r*. *rhombeatus*).

In contrast to males, females showed higher overlap in the non-breeding season than the breeding season (Figs 1 & 2). Although only four female-female non-breeding season dyads met our criteria for inclusion in our analyses, another three Brevard females monitored during the non-breeding seasons of 1999 and 2000 had relatively high home range overlap (median VI = 0.09 and 0.35 for core area and home ranges, respectively, e.g., Fig 4F). In contrast, home range overlap among these same three females during the breeding season was lower (median VI = 0.06 and 0.18 for core area and home ranges, respectively, e.g., Fig 4E), a pattern consistent with the results of our analyses. Cottone and Bauer [20] did not observe breeding season home range overlap in female rhombic skaapstekers. Low levels of female home range overlap may reflect efforts to minimize competition for food so that females can secure sufficient resources for reproduction [6]. Yet if this was the case in our system we would expect female-female home range overlap to be lowest during the non-breeding season when temperatures are warmer and most foraging occurs [40]. Whitaker and Shine [24] found that female brownsnakes (*Pseudonaja textilis*) often cohabited burrows while males were never observed to do so, a pattern analogous to that seen by Webb et al. [28] with regard to small-eyed snakes (*Cryptophis nigrescens*) using shelter rocks. However, we note that spatial overlap does not imply the lack of temporal avoidance [23,24].

Studies reporting low home range overlap in snakes are a minority. It is difficult to make direct comparisons of home range overlap among studies because of differences in home range estimators and methods used to calculate overlap. For example, most snake studies estimated home ranges using minimum convex polygons (MCP) instead of fixed kernels even though the latter provides a probabilistic representation of space use that provides a more accurate measure of spatial overlap [54]. Nevertheless, we suggest that qualitative comparisons are still possible, particularly of factors influencing home range overlap (e.g., sex, season). Of 19 published studies that addressed home range overlap in snakes, 15 (79%) inferred high levels of home range overlap (e.g., non-exclusive home ranges). However, two of these studies found that, despite broad home range overlap (11–89% MCP overlap), snakes avoided using shelter sites that were or had previously been occupied by conspecifics [23,24]. Seven of these 15 studies quantified the degree of home range overlap either as a percentage of the home range overlapped by one or more individuals [17,19,23,24,64] or the number overlapping home ranges [40,65]. For example, Hyslop et al. [40] reported that *D*. *couperi* home ranges in southern Georgia were overlapped by the annual home ranges of at least six other individuals. Mitrovich et al. [19] calculated the proportion of an individual’s home range overlapped by other individuals for coachwhips (*Coluber flagellum fuliginosus*) in southern California and found that mean overlap was 0.49–0.89 across three sites. Eight studies that did not quantify the degree of home range overlap reported overlap as “substantial” or “extensive” [18,66,67,68,69,70,71,72]. Anguiano and Diffendorfer [17] found that female California kingsnakes shared a greater percentage of their MCP home range with males (mean = 63%) than males did with females (19%) or other males (27%). However, males never shared core areas. In contrast, “extensive” home range overlap was reported between male and female carpet pythons (*Morelia spilota*, [67,70]) and green pythons (*Morelia viridis*, [64]).

Patterns of low spatial overlap among males combined with relatively higher inter-sexual spatial overlap are seen in many mammalian carnivores [10,11,73,74]. Powell [11,75] hypothesized that high spatial overlap between males and females in a species where males search for females could confer a net advantage to males by providing easy access to females even if breeding occurs seasonally. Low year-round home range overlap among male *D*. *couperi* combined with high male-female overlap may therefore act to increase male reproductive success. While high levels of male-female overlap outside of the breeding season could lead to intraspecific competition for food resources, the advantages of such overlap to males may exceed the costs [19, 66]. Powell [11,75] hypothesized that, in species with male-biased sexual-size dimorphism, males may force spatial overlap on females. Additionally, the costs of inter-sexual home range overlap are expected to decrease if male foraging movements within their home range avoid widespread behavioral or numerical suppression of prey [11,75]. The patterns of *D*. *couperi* spatial overlap we observed are consistent with these hypotheses, particularly since *D*. *couperi* males are larger than females [38]. In our study, males also maintained larger home ranges than females during both the breeding and non-breeding seasons [38] which could serve as a means to reduce inter-sexual competition. However, explicitly testing these hypotheses requires information on mating success and the degree of dietary overlap between males and females.

Resource abundance and competition may also influence spatial overlap. Low levels of spatial overlap are theoretically most beneficial when resource abundance and availability is neither extremely low nor extremely high, because in each instance the costs of excluding conspecifics may exceed the benefits [32,76]. While data from mammalian carnivores often supports this hypothesis [8,77,78], it is less clear if this pattern should hold for snakes because, as ectotherms, they have lower energetic requirements, greater conversion efficiencies, are able to consume larger meals, and are more resilient to fasting than similarly-sized endotherms [79,80,81]. However, snake species with larger body sizes, home ranges, and more active foraging strategies generally have higher energetic requirements [71,82,83,84,85,86,87], which may make exclusive use of foraging habitats more advantageous if it reduces intraspecific competition for prey. While data on *D*. *couperi* energetic requirements are unavailable, *D*. *couperi* is a large (> 2 m SVL), actively foraging species with some of the longest daily movement distances and home range sizes reported for snakes [38,39,40]. The relatively low levels of spatial overlap observed in our study are consistent with the hypothesis that reduced spatial overlap is more advantageous for snake species with higher energetic requirements. While high home range overlap has been reported in ambush foragers [65,70] or species with small home ranges (< 25 ha, [17,18,23,64], other active foraging species of snakes also show high levels of spatial overlap [19,68,71]. Additionally, Hyslop et al. [40] found broad overlap among *D*. *couperi* home ranges in southern Georgia although they did not specify how much of this overlap was due to shared use of overwintering habitat or migration routes. It is therefore less clear how energetic requirements may influence home range overlap in snakes.

Alternatively, low spatial overlap may be driven by a “bet-hedging” strategy where exclusive home ranges are maintained despite temporal fluctuations in prey abundance [78,88,89,90]. Jenkins [90] hypothesized that such a strategy might explain inter-annual fidelity in western rattlesnake (*Crotalus oreganus*) summer home ranges despite changing prey availability. Similarly, *D*. *couperi* appear to show inter-annual fidelity to non-breeding season home ranges within our study area [38]. However, additional data on energetics and variation in prey availability are needed to test these alternative hypotheses. Another factor potentially contributing to low spatial overlap in *D*. *couperi* is the potential for cannibalism. Snakes comprise a major portion of *D*. *couperi* diets and cannibalism has been documented [37,91]. However, if individuals avoided conspecifics to reduce the threat of cannibalism, we would then expect very little male-female overlap because males are larger and more able to prey on females than other males.

We acknowledge that our study suffers from the limitation of not simultaneously monitoring all individuals within our study areas. The presence of non-telemetered individuals could result in greater spatial overlap among individuals than we observed. *Drymarchon couperi* in our study areas are difficult to detect, which makes it difficult to assess the degree of bias in our results. However, non-telemetered snakes and telemetered snakes that did not meet the criteria for inclusion in our analyses still displayed behaviors consistent with our results. For example, in our Highlands study, we captured nine unmarked adult males overlapping the home ranges of telemetered males. Although this may suggest higher rates of male-male home range overlap than our results indicate, seven of these captures (78%) occurred during the breeding season when males are most likely to overlap spatially. We implanted transmitters in four of the nine snakes and found that their home ranges overlapped little with simultaneously adjacent telemetered males during the subsequent non-breeding season (e.g., Fig 4A & 4B). Patterns of home range overlap among Brevard snakes not included in our analyses were also consistent with our results. While anecdotal, we suggest that these observations lend confidence to the results of our analyses.

Landscape composition could influence conspecific overlap, although our sample sizes did not permit us to examine this factor. Developed landscapes may compress home ranges and inflate population densities relative to less-disturbed landscapes [92], potentially leading to greater levels of home range overlap. This pattern was observed in coachwhips in isolated habitat fragments [19]. Breininger et al. [39] found that *D*. *couperi* home ranges were smaller for both sexes in developed landscapes. This may have confounded the degree of spatial overlap among the Brevard snakes because many were monitored in developed landscapes. One of our two broadest overlapping male-male dyads (non-breeding season, VI = 0.37) was in an urbanized landscape. However, we observed another broadly overlapping male-male dyad in an undeveloped landscape (breeding season, VI = 0.37). Furthermore, urban red foxes (*Vulpes vulpes*) maintained exclusive home ranges similar to those of rural foxes despite continuous shifts in the urban home ranges, possibly caused by fluctuating food resources [93].

We also note that our study did not examine the behavioral mechanisms responsible for maintaining the observed levels of spatial overlap. The mechanisms responsible for spatial segregation are diverse and include antagonistic physical interactions, physical or auditory display, or passive actions such as scent marking [12,13,94,95]. We think it is unlikely that *D*. *couperi* engage in active defense of their home ranges as we did not observe movement patterns consistent with territoriality, such as patrolling the edge of the home range [95]. Nor did we observe antagonistic interactions outside of the breeding season suggestive of territorial defense. Nevertheless, exclusive space use may be maintained passively through scent markings (e.g., [12]). Snakes have excellent olfactory capabilities that are used in foraging [96,97,98], mate selection [99], and refuge selection [100,101]. Furthermore, multiple studies have demonstrated that snakes can obtain information about conspecific body size from scent [101,102], which could allow smaller, subordinate individuals to avoid areas occupied by larger, dominant individuals [101]. It is possible that the within-home range movements we observed were sufficient to maintain scent markings that conspecifics could detect. Alternatively, individuals may retain some spatial memory of areas used by conspecifics and avoid those areas. Investigating the mechanisms responsible for maintaining reduced spatial overlap in snakes could provide greater insights into the costs and benefits of maintaining reduced overlap. This could in turn contribute to a more comprehensive understanding of snake social systems and how they contrast with those of ecologically comparable terrestrial vertebrates (e.g., small- to medium-sized mammalian carnivores).

## References

[1] MacDonald DW, Mosser A, Gittleman JL (2010) Felid society In: MacDonald DW, Loveridge AJ, editors. Biology and conservation of wild felids. Oxford: Oxford University Press Inc pp. 126–160.

[2] Owen-Smith N (1977) On territoriality in ungulates and an evolutionary model. Q Rev Biol 52: 1–38.

[3] Wolff JO (1997) Population regulation in mammals: an evolutionary perspective. J Anim Ecol 66: 1–13.

[4] Fryxell JM, Falls JB, Falls EA, Brooks RJ, Dix L, Strickland MA (1999) Density dependence, prey dependence, and population dynamics of martens in Ontario. Ecol 80: 1311–1321.

[5] Rogers LL (1987) Effects of food-supply and kinship on social behavior, movements, and population growth of black bears in northeastern Minnesota. Wildl Monogr 97: 1–72.

[6] Ostfeld RS (1990) The ecology of territoriality in small mammals. Trends in Ecology & Evolution 5: 411–415. 21232403 10.1016/0169-5347(90)90026-A

[7] Gehrt SD, Fritzell EK (1998) Resource distribution, female home range dispersion and male spatial interactions: group structure in a solitary carnivore. Animal Behaviour 55: 1211–1227. 9632506 10.1006/anbe.1997.0657

[8] McLoughlin PD, Ferguson SH, Messier F (2000) Intraspecific variation in home range overlap with habitat quality: A comparison among brown bear populations. Evol Ecol 14: 39–60.

[9] Maher CR, Lott DF (1995) Defininitions of territoriality used in the study of variation in vertebrate spacin systems. Anim Behav 49: 1581–1597.

[10] Powell RA (1979) Mustelid spacing patterns—variations on a theme by *Mustella*. J Comp Ethol 50: 15–3165.

[11] Powell RA (1994) Structure and spacing of *Martes* populations In: Buskirk SW, Harestad AS, Raphael MG, Powell RA, editors. Martens, sables, and fishers: biology and conservation. Ithaca: Cornell University Press pp. 101–121.

[12] Gese EM (2001) Territorial defense by coyotes (*Canis latrans*) in Yellowstone National Park, Wyoming: who, how, where, when, and why. Can J Zool 79: 980–987.

[13] Smith CC (1968) Adaptive nature of social organization in genus of 3 squirrels *Tamiasciurus*. Ecol Monogr 38: 31–64.

[14] Ostfeld RS (1986) Territoriality and mating system of California voles. J Anim Ecol 55: 691–706.

[15] Brown JL (1969) Territorial behavior and population regulation in birds: a review and re-evaluation. Wilson Bull 81: 293–329.

[16] Stamps JA (1983) Sexual selection, sexual dimorphism, and territoriality In: Huey RB, Pianka ER, Schoener TW, editors. Lizard ecology: studies of a model organism. Cambridge: Harvard University Press pp. 169–204.

[17] Anguiano MP, Diffendorfer JE (2015) Effects of fragmentation on the spatial ecology of the California kingsnake (*Lampropeltis californiae*). J Herpetol 49: 420–427.

[18] Diffendorfer JE, Rochester C, Fisher RN, Brown TK (2005) Movement and space use by Coastal Rosy Boas (*Lichanura trivirgata roseofusca*) in Coastal Southern California. J Herpetol 39: 24–36.

[19] Mitrovich MJ, Diffendorfer JE, Fisher RN (2009) Behavioral response of the coachwhip (*Masticophis flagellum*) to habitat fragment size and isolation in an urban landscape. J Herpetol 43: 646–656.

[20] Cottone AM, Bauer AM (2013) The vernal spatial ecology and mating behaviors of the rhombic skaapsteker, *Psammophylax rhombeatus rhombeatus* (Serpentes: Psammophiidae), from the Western Cape, South Africa. Copeia 2013: 194–200.

[21] Webb JK, Shine R (1997) A field study of the spatial ecology and movements of a threatened snake species, *Hoplocephalus bungaroides*. Biol Conserv 82: 203–217.

[22] Steen DA, Smith LL (2009) Eastern kingsnake (*Lampropeltis getula getula*) home ranges exhibit limited overlap. Southeast Nat 8: 553–558.

[23] Fitzgerald M, Shine R, Lemckert F (2002) Spatial ecology of arboreal snakes (*Hoplocephalus stephensii*, Elapidae) in an eastern Australian forest. Austral Ecol 27: 537–545.

[24] Whitaker PB, Shine R (2003) A radiotelemetric study of movements and shelter-site selection by free-ranging brownsnakes (*Pseudonaja textilis*, Elapidae). Herpetol Monogr 17: 130–144.

[25] Gregory PT, Macartney JM, Larsen KW (1987) Spatial patterns and movements In: Seigel RA, Collins JT, Novak SS, editors. Snakes: ecology and evolutionary biology. New York: Macmillan Publishing Company pp. 366–395.

[26] Greene HW (1997) Snakes: the evolution of mystery in nature. Berkeley: University of California Press 365 pp.

[27] Huang W-S, Greene HW, Chang T-J, Shine R (2011) Territoriality behavior in Taiwanese kukrisnakes (*Oligodon formosanus*). Proc Nat Acad Sci 108: 7455–7459. 10.1073/pnas.1101804108 21502515 PMC3088593

[28] Webb JK, Scott ML, Whiting MJ, Shine R (2015) Territoriality in a snake. Behav Ecol Sociobiol 69: 1657–1661.

[29] Gregory PT (1984) Communal denning in snakes In: Seigel RA, Hunt LE, Knight JL, Malaret L, Zushlag NL, editors. Vertebrate ecology and systematics—a tribute to Henry S Fitch. Lawrence: Museum of Natural History, The University of Kansas pp. 57–75.

[30] Gillingham JC (1987) Social behavior In: Seigel RA, Collins JT, Novak SS, editors. Snakes: ecology and evolutionary biology. New York: MacMillan Publishing Company pp. 184–209.

[31] Graves BM, Duvall D (1995) Aggregation of Squamate reptiles associated with gestation, oviposition, and parturition. Herpetol Monogr 9: 102–119.

[32] Maher CR, Lott DF (2000) A review of ecological determinants of territoriality within vertebrate species. Am Midl Nat 143: 1–29.

[33] Conant R, Collins JT (1998) A field guide to reptiles and amphibians: eastern and central North America. New York: Houghton Mifflin Company 640 pp.

[34] Stevenson DJ, Enge KM, Carlile LD, Dyer KJ, Norton TM, Hyslop NL, et al (2009) An eastern indigo snake (*Drymarchon couperi*) mark-recapture study in southeastern Georgia. Herpetol Conserv Biol 4: 30–42.

[35] Enge KM, Stevenson DJ, Elliot MJ, Bauder JM (2013) The historical and current distribution of the eastern indigo snake (*Drymarchon couperi*). Herpetol Conserv Biol 8: 288–307.

[36] Hyslop NL (2007) Movements, habitat use, and survival of the threatened eastern indigo snake (Drymarchon couperi) in Georgia. PhD dissertation, University of Georgia.

[37] Stevenson DJ, Bolt MR, Smith DJ, Enge KM, Hyslop NL, Norton TM, et al (2010) Prey records for the eastern indigo snake (*Drymarchon couperi*). Southeast Nat 9: 1–18.

[38] Bauder JM, Breininger DR, Bolt MR, Legare ML, Jenkins CL, Rothermel BB, et al (In Press) Seasonal variation in eastern indigo snake (*Drymarchon couperi*) movement patterns and space use in peninsular Florida at multiple temporal scales. Herpetologica 72.

[39] Breininger DR, Bolt MR, Legare ML, Drese JH, Stolen ED (2011) Factors influencing home-range sizes of eastern indigo snakes in central Florida. J Herpetol 45: 484–490.

[40] Hyslop NL, Meyers JM, Cooper RJ, Stevenson DJ (2014) Effects of body size and sex of *Drymarchon couperi* (Eastern Indigo Snake) on habitat use, movements, and home range size in Georgia. J Wildl Manage 78: 101–111.

[41] Stevenson DJ, Dyer KJ, Willis-Stevenson BA (2003) Survey and monitoring of the eastern indigo snake in Georgia. Southeast Nat 2: 393–408.

[42] Hyslop NL, Cooper RJ, Meyers JM (2009) Seasonal shifts in shelter and microhabitat use of *Drymarchon couperi* (Eastern Indigo Snake) in Georgia. Copeia 2009: 458–464.

[43] Bauder JM, Barnhart P (2014) Factors affecting the accuracy and precision of triangulated radio telemetry locations of Eastern Indigo Snakes (*Drymarchon couperi*). Herpetol Rev 45: 590–597.

[44] Abrahamson WG, Johnson AF, Layne JN, Peroni PA (1984) Vegetation of the Archbold Biological Station, Florida: an example of the southern Lake Wales Ridge. Florida Sci 47: 209–250.

[45] Layne JN, Steiner TM (1996) Eastern indigo snake (Drymarchon corais couperi): summary of research conducted on Archbold Biological Station. Report prepared under order 43910-6-0134 to the US Fish and Wildlife Service. Jackson, Mississippi.

[46] Moser BW, Garton EO (2007) Effects of telemetry location error on space-use estimates using a fixed kernel density estimator. J Wildl Manage 71: 2421–2426.

[47] Bauder JM, Breininger DR, Bolt MR, Legare ML, Jenkins CL, McGarigal K. (2015) The role of the bandwidth matrix in influencing kernel home range estimates for snakes using VHF telemetry data. Wildl Res 42: 437–453.

[48] Duong T, Hazelton ML (2003) Plug-in bandwidth matrices for bivariate kernel density estimation. J Nonparametr Stat 15: 17–30.

[49] Duong T (2007) ks: kernel density estimation and kernel discriminant analysis for multivariate data in R. J Stat Soft 21: 1–16.

[50] Duong T (2014) ks: kernel smoothing. R package version 1.9.3 http://CRANR-projectorg/package=ks.

[51] Harris S, Cresswell WJ, Forde PG, Trewhella WJ, Woollard T, Wray S (1990) Home-range analysis using radio-tracking data—a review of problems and techniques particularly as applied to they study of mammals. Mammal Rev 20: 97–123.

[52] Laver PN, Kelly MJ (2008) A critical review of home range studies. J Wildl Manage 72: 290–298.

[53] Borger L, Franconi N, De Michele G, Gantz A, Meschi F, Manica A, et al (2006) Effects of sampling regime on the mean and variance of home range size estimates. J Anim Ecol 75: 1393–1405. 17032372 10.1111/j.1365-2656.2006.01164.x

[54] Fieberg J, Kochanny CO (2005) Quantifying home-range overlap: The importance of the utilization distribution. J Wildl Manage 69: 1346–1359.

[55] Fieberg J (2014) Home range overlap indices implemented using kernel density estimators with plug-in smoothing parameters and Program R. University of Minnesota Digital Conservancy. Retrieved from http://hdl.handle.net/11299/163012.

[56] Anderson MJ (2001) A new method for non-parametric multivariate analysis of variance. Austral Ecol 26: 32–46.

[57] McArdle BH, Anderson MJ (2001) Fitting multivariate models to community data: A comment on distance-based redundancy analysis. Ecol 82: 290–297.

[58] Oksanen J, Blanchet FG, Kindt R, Legendre P, Minchin PR, O'Hara RB, et al. (2015) vegan: community ecology package. R package version 2.2–1 http://CRANR-projectorg/package=vegan.

[59] Holm S (1979) A simple sequentially rejective multiple test procedure. Scand J Stat 6: 65–70.

[60] Southworth H, Heffernan JE (2013) texmex: statistical modeling of extreme values. R package version 2.1 http://CRANR-projectorg/package=texmex.

[61] Burnham KP, Anderson DR (2002) Model selection and multimodel inference. New York: Springer 488 pp.

[62] Hyslop NL, Meyers JM, Cooper RJ, Norton TM (2009) Survival of radio-implanted *Drymarchon couperi* (eastern indigo snake) in relation to body size and sex. Herpetologica 65: 199–206.

[63] Speake DW, McGlincy D, Smith C (1987) Captive breeding and experimental reintroducion of the eastern indigo snake. Proceedings of the Third Southeastern Nongame and Endangered Wildlife Symposium 3: 84–90.

[64] Wilson D, Heinsohn R, Legge S (2006) Age- and sex-related differences in the spatial ecology of a dichromatic tropical python (*Morelia viridis*). Austral Ecol 31: 577–587.

[65] Secor SM (1994) Ecological significane of movements and activity range for the sidewinder, *Crotalus cerastes*. Copeia 1994: 631–645.

[66] Weatherhead PJ, Hoysak DJ (1989) Spatial and activity patterns of black rat snakes (*Elaphe obsoleta*) from radiotelemetry and recapture data. Can J Zool 67: 463–468.

[67] Slip DJ, Shine R (1988) Habitat use, movemens and activity patterns of free-ranging diamond pythons, *Morelia spilota spilota* (Serpentes, Boidae)—a radiotelemetric study. Aust Wildl Res 15: 515–531.

[68] Plummer MV, Congdon JD (1994) radiotelemetric study of activity and movements of racers (*Coluber constrictor*) associated with a Carolina bay in South Carolina. Copeia 1994: 20–26.

[69] Blouin-Demers G, Weatherhead PJ (2002) Implications of movement patterns for gene flow in black rat snakes (*Elaphe obsoleta*). Can J Zool 80: 1162–1172.

[70] Pearson D, Shine R, Williams A (2005) Spatial ecology of a threatened python (*Morelia spilota imbricata*) and the effects of anthropogenic habitat change. Austral Ecol 30: 261–274.

[71] Carfagno GLF, Weatherhead PJ (2008) Energetics and space use: intraspecific and interspecific comparisons of movements and home ranges of two Colubrid snakes. J Anim Ecol 77: 416–424. 10.1111/j.1365-2656.2008.01342.x 18254921

[72] Corey B, Doody JS (2010) Anthropogenic influences on the spatial ecology of a semi-arid python. J Zool 281: 293–302.

[73] Sandell M (1989) The mating tactics and spacing patterns of solitary carnivores In: Gittleman JL, editor. Carnivore behavior, ecology, and evolution. Ithaca: Cornell University Press pp. 164–182.

[74] Ferreras P, Beltran JF, Aldama JJ, Delibes M (1997) Spatial organization and land tenure system of the endangered Iberian lynx (*Lynx pardinus*). J Zool 243: 163–189.

[75] Powell RA (1993) Why do some forest carnivores exhibit intrasexual territoriality and what are the consequences for management? Proc Int Union Game Biol 21: 268–273.

[76] Carpenter FL, MacMillen RE (1976) Threshold model of feeding territoriality and test with a Hawaiian Honeycreeper. Science 194: 639–642. 17818435 10.1126/science.194.4265.639

[77] Eide NE, Jepsen JU, Prestrud P (2004) Spatial organization of reproductive Arctic foxes *Alopex lagopus*: responses to changes in spatial and temporal availability of prey. J Anim Ecol 73: 1056–1068.

[78] Lopez-Bao JV, Rodriguez A, Delibes M, Fedriani JM, Calzada J, Ferreras P, et al (2014) Revisiting food-based models of territoriality in solitary predators. J Anim Ecol 83: 934–942. 10.1111/1365-2656.12226 24720673

[79] Pough FH (1980) Advantages of ectotherm for tetrapods. Am Nat 115: 92–112.

[80] McCue MD (2007) Western diamondback rattlesnakes demonstrate physiological and biochemical strategies for tolerating prolonged starvation. Physiol Biochem Zool 80: 25–34. 17160877 10.1086/509057

[81] Nowak EM, Theimer TC, Schuett GW (2008) Functional and numerical responses of predators: where do vipers fit in the traditional paradigms? Biol Rev 83: 601–620. 10.1111/j.1469-185X.2008.00056.x 18947336

[82] Ruben JA (1976) Aerobic and anaerobic metabolism during activity in snakes. J Comp Physiol 109: 147–157.

[83] Secor SM, Nagy KA (1994) Bioenergetic correlates of foraging mode for the snakes *Crotalus cerastes* and *Masticophis flagellum*. Ecol 75: 1600–1614.

[84] Plummer MV, Congdon JD (1996) Rates of metabolism and water flux in free-ranging racers, *Coluber constrictor*. Copeia 1996: 8–14.

[85] Nagy KA (2005) Field metabolic rate and body size. J Exp Biol 208: 1621–1625. 15855393 10.1242/jeb.01553

[86] Lelievre H, Le Henanff M, Blouin-Demers G, Naulleau G, Lourdais O (2010) Thermal strategies and energetics in two sympatric colubrid snakes with contrasted exposure. J Comp Physiol B 180: 415–425. 10.1007/s00360-009-0423-8 20091170

[87] Lelievre H, Moreau C, Blouin-Demers G, Bonnet X, Lourdais O (2012) Two syntopic Colubrid snakes differ in their energetic requiremens and in their use of space. Herpetologica 68: 358–364.

[88] White PJ, Ralls K (1993) Reproduction and spacing patterns of kit foxes relative to changing prey availability. J Wildl Manage 57: 861–867.

[89] Bateson M (2002) Recent advances in our understanding of risk-sensitive foraging preferences. Proc Nutr Soc 61: 509–516. 12691180 10.1079/pns2002181

[90] Jenkins CL (2007) Ecology and conservation of rattlesnakes in sagebrush steppe ecosystems: landscape disturbance, small mammal communities, and Great Basin rattlesnake reproduction. PhD dissertation, Idaho State University.

[91] Smith CR (1987) Ecology of juvenile and gravid Eastern Indigo Snakes in north Florida. MS thesis, Auburn University.

[92] Salek M, Drahnikova L, Tkadlec E (2015) Changes in home range sizes and population densities of carnivore species along the natural to urban habitat gradient. Mammal Rev 45: 1–14.

[93] Doncaster CP, MacDonald DW (1991) Drifting territoriality in the red fox *Vulpes vulpes*. J Anim Ecol 60: 423–439.

[94] Wronski T, Apio A, Baranga J, Plath M (2006) Scent marking and territorial defence in male bushbuck (*Tragelaphus scriptus*). J Zool 270: 49–56.

[95] Giuggioli L, Potts JR, Harris S (2011) Animal interactions and the emergence of territoriality. PLoS One 7: e1002008. 10.1371/journal.pcbi.1002008 PMC3053310 21423708

[96] Duvall D, Chiszar D, Hayes WK, Leonhardt JK, Goode MJ (1990) Chemical and behavioral ecology of foraging in prairie rattlesnakes (*Crotalus viridis viridis*). J Chem Ecol 16: 87–101. 10.1007/BF01021270 24264898

[97] Theodoratus DH, Chiszar D (2000) Habitat selection and prey odor in the foraging behavior of western rattlesnakes (*Crotalus viridis*). Behav 137: 119–135.

[98] Clark RW (2004) Timber rattlesnakes (*Crotalus horridus*) use chemical cues to select ambush sites. J Chem Ecol 30: 607–617. 15139311 10.1023/b:joec.0000018632.27010.1e

[99] LeMaster MP, Moore IT, Mason RT (2001) Conspecific trailing behaviour of red-sided garter snakes, *Thamnophis sirtalis parietalis*, in the natural environment. Anim Behav 61: 827–833.

[100] Reinert HK, Zappalorti RT (1988) Field observation of the association of adult and neonatal timber rattlesnakes, *Crotalus horridus*, with possible evidence for conspecific trailing. Copeia: 1057–1059.

[101] Scott ML, Whiting MJ, Webb JK, Shine R (2013) Chemosensory discrimination of social cues mediates space use in snakes, *Cryptophis nigrescens* (Elapidae). Anim Behav 85: 1493–1500.

[102] Shine R, Phillips B, Waye H, LeMaster M, Mason RT (2003) Chemosensory cues allow courting male garter snakes to assess body length and body condition of potential mates. Behav Ecol Sociobiol 54: 162–166.

